# Cost-effectiveness analysis of current non-mandatory hepatitis B vaccination coverage vs expanding coverage among healthcare workers in Ethiopia

**DOI:** 10.1186/s40545-022-00458-4

**Published:** 2022-10-17

**Authors:** Dinksew Tewuhibo, Getahun Asmamaw, Wondim Ayenew

**Affiliations:** 1 Department of Pharmacy, Madda Walabu University, Bale Robe, Ethiopia; 2 Department of Pharmacy, Arba Minch University, Arba Minch, Ethiopia; 3 Department of Pharmaceutics, University of Gondar, Gondar, Ethiopia

**Keywords:** Cost-effectiveness, Hepatitis-B virus prevalence, Hepatitis-B vaccination coverage, Healthcare workforce, Ethiopia

## Abstract

**Background:**

Ethiopia is a country with high endemicity in Hepatitis B (HepB) virus infection. However, only 14% of healthcare workers (HCWs) are currently immunized via a non-mandatory strategy in the country. Hence, this study aimed to estimate the cost-effectiveness of the current vaccination coverage and increasing coverage among HCWs in Ethiopia.

**Methods:**

Based on current practice, the study considered a monovalent HepB vaccine, which has a 90% protection rate with a complete three-dose series for lifelong protection. Markov model for current coverage (14%) and expanding vaccination coverage to 80% (as per World Health Organization (WHO) recommendation) was simulated based on the data got from both primary and secondary data. Secondary data, particularly cost and effectiveness data, were gained from published articles, WHO guidelines, and Ethiopian Federal Ministry of Health documents. Cost-related data for vaccination and chronic HepB treatment were also gathered by interviewing expertise from Tikur Anbesa specialized hospital. We conducted the study from a healthcare payer perspective, with a 3% discount rate of cost and health outcome as recommended by the WHO. The primary health outcome was measured by the Incremental Cost-Effectiveness Ratio (ICER). We employed deterministic analysis and tornado diagrams to manage parameter uncertainty and show a plausible range of cost and effectiveness of variables.

**Results:**

Current vaccination program is more expensive (USD 29.99) with a more additional cost of USD 1.32 and with reduced effectiveness of 0.08 Life Years (LYs) compared to the expanded HepB vaccination strategy which costs USD 28.67 and gives a relatively high total LY gain of 28.62. The resulting ICER was USD-16.23 per LYs gained. The negative ICER shows that the expanded HepB vaccination strategy dominated the current vaccination strategy. A one-way sensitivity analysis also revealed that the current vaccine coverage was dominated by an increase in the risk of infection among unvaccinated individuals.

**Conclusions:**

Expanded vaccination coverage (to 80%) was found the most cost-effective strategy in Ethiopian HCWs compared to the current non-mandatory vaccine coverage (14%). In addition, the results of one-way and two-way sensitivity analysis reveal the robustness of our model conclusion.

## Background

Hepatitis B Virus (HBV) is one of the serious global health burdens that affects the lives of over two billion people [1, 2]. It is primarily transmitted through percutaneous or mucosal contact with infected blood and body fluids, and through occupational exposure of healthcare workers (HCWs) during dental, medical, and surgical procedures [1]. HBV could be acute or chronic based on the persistence of Hepatitis B (HepB) surface antigen (HBsAg) for less or over 6 months [3]. Most of acute HepB infections are self-limiting and do not require treatment. However, 0.5–1% of people could develop fulminant hepatitis, which has a mortality rate of 70–80% [4]. Meanwhile, 3–5% of adults with acute HepB have a chance of progressing into Chronic Hepatitis B (CHB) [5]. Patients with CHB have a 40% risk of developing sequelae such as cirrhosis, liver failure, and Hepatocellular Carcinoma [3]. Thus, 5–25% of people with CHB infection die each year because of liver complications [6]. Despite this, only 5% of CHB patients have access to treatment in Ethiopia for a variety of reasons, including a lack of finance, a scarcity of molecular diagnostic tests, regulatory limits on antiviral medications, and a lack of commitment from policymakers [6, 7].

WHO recently estimated that over 350 million people globally are chronic carriers of the virus [8]. The endemicity of HBV varies across the globe, with Asia and Africa having the highest prevalence [9]. HCWs, in particular, are ten times more likely than the general population to acquire HBV [10]. The annual global incidence of HepB infection in HCWs is approximately 6% [8, 11]. Similarly, a high risk of exposure was reported among physicians, clinical nurses, midwives, laboratory technicians, and anesthetists in Ethiopia [12, 13]. Existing evidence also supports the high prevalence of HepB among HCWs in Ethiopia [14]. For example, at Gondar and Addis Ababa referral hospitals, the seroprevalence of HepB viral infection was 4.52% and 2.6%, respectively [12, 13]. Predisposing factors such as contamination because of medical procedures and work overload are majorly claimed to be responsible [13, 15–17]. This implies that the burden of this viral infection is an additional challenge for Ethiopia's healthcare system, which has been facing infectious and non-communicable diseases [14].

Three doses of monovalent HepB vaccinations given every 6 months are currently the most effective and safe strategy to prevent HepB infection [18]. WHO and the Ethiopian Federal Ministry of Health (FMOH) both encourage that all HCWs be vaccinated against HBV [5, 14]. These institutions have aimed to achieve HBV vaccine coverage of at least 80% among HCWs [19, 20]. However, HBV vaccine coverage among the entire healthcare workforce who directly or indirectly participate in healthcare service delivery, including health extension workers, medical waste handlers, and janitors, has remained low in several countries (< 20%) [21]. Similarly, the minimal (14%) coverage of HepB vaccination among healthcare professionals was also reported in Ethiopia [13, 17]. Unfortunately, a non-mandatory strategy is being used for vaccination against HBV in HCWs in the country [13, 17]. Hence, this cost-effectiveness analysis determined the cost averted and disease prevention (life year gains) of increasing HepB vaccine coverage among all healthcare workforces such as nurses, laboratory, pharmacy, medical doctors, midwives, janitors, supportive staff compared to current non-mandatory HepB vaccination coverage (14%).

## Methods

### Study population and design

Ethiopian FMOH is the primary public healthcare provider, in charge of policy formation, planning, development, and management of all health issues. The minister's office employs around 241,250 people in the health sector, including health extension workers (15%), paramedical professionals (35%), medical doctor professionals (3%), supportive and administrative personnel (30%), and others [20]. In this report, the average age of HCWs is 32 years, which was considered in the analysis. This study included all Ethiopian HCWs because of their higher occupational exposure to HBV than the general population [12]. All healthcare facilities in the country, including 16,243 health posts, 3,743 health centers, 220 primary hospitals, 64 general hospitals, 27 referral hospitals, and other administrative offices such as the regional health bureau, district health offices, and FMOH were also considered [20]. The study was conducted from the healthcare payer perspective (Ethiopian FMOH) (Table 1). Furthermore, since it was assumed that vaccines offer lifetime protection, the cost of expanding vaccination coverage, impact on LY gains, and ICER were assessed over the 65-year lifetime of HCWs. Therefore, the cost and LYs were adjusted to a 3% discount rate as of WHO recommendation (Table 1). Accordingly, a synthesis-based estimate of the data was used, references to many studies from China, the USA, and Ethiopia were made, and a mean value was taken to correct misleading data findings from a single study [1, 5, 8, 15, 22].

**Table 1:** Method summary of economic evaluations of cost-effectiveness analysis of expanding hepatitis B vaccination among the healthcare workforce in Ethiopia

Disease	Hepatitis B
Population	Healthcare workforce
Intervention	Increasing vaccination
Comparators	Current vaccination
Outcome	Disease prevented, QALY gained, Cost
Perspective	Healthcare payer
Time Horizon	Lifetime
Discounting	3%
Sensitivity Analysis	Deterministic Sensitivity analysis
Type of Model	Markov modeling

All necessary pharmaceuticals, including the HepB vaccine, were procured and supplied primarily by the Ethiopian Pharmaceutical Supply Agency (EPSA). EPSA is an institution in providing pharmaceuticals, laboratory reagents, medical equipment, and supplies to the Ethiopian people (direct supplier to healthcare facilities in the country) [23]. Antiviral drugs such as Tenofovir 300 mg and Lamivudine 150 mg which are used to treat of CHB are not included in the pharmaceutical procurement list of Ethiopia [24]. However, they are assumed to be supplied by EPSA and are currently available at Tikur Anbesa specialized Hospital (TASH); the largest referral hospital in the country. Cost-related data were gathered by interviewing experts from TASH (Nurses, physicians, and Pharmacists), and WHO HBV guidelines. Effectiveness-related information was also gathered from distinctive pieces of literature review. All costs were expressed in United States dollars (USD) at an exchange rate of 28 Ethiopian Birr to USD l on June 11, 2019.

### Model structure

**Fig. 1:**
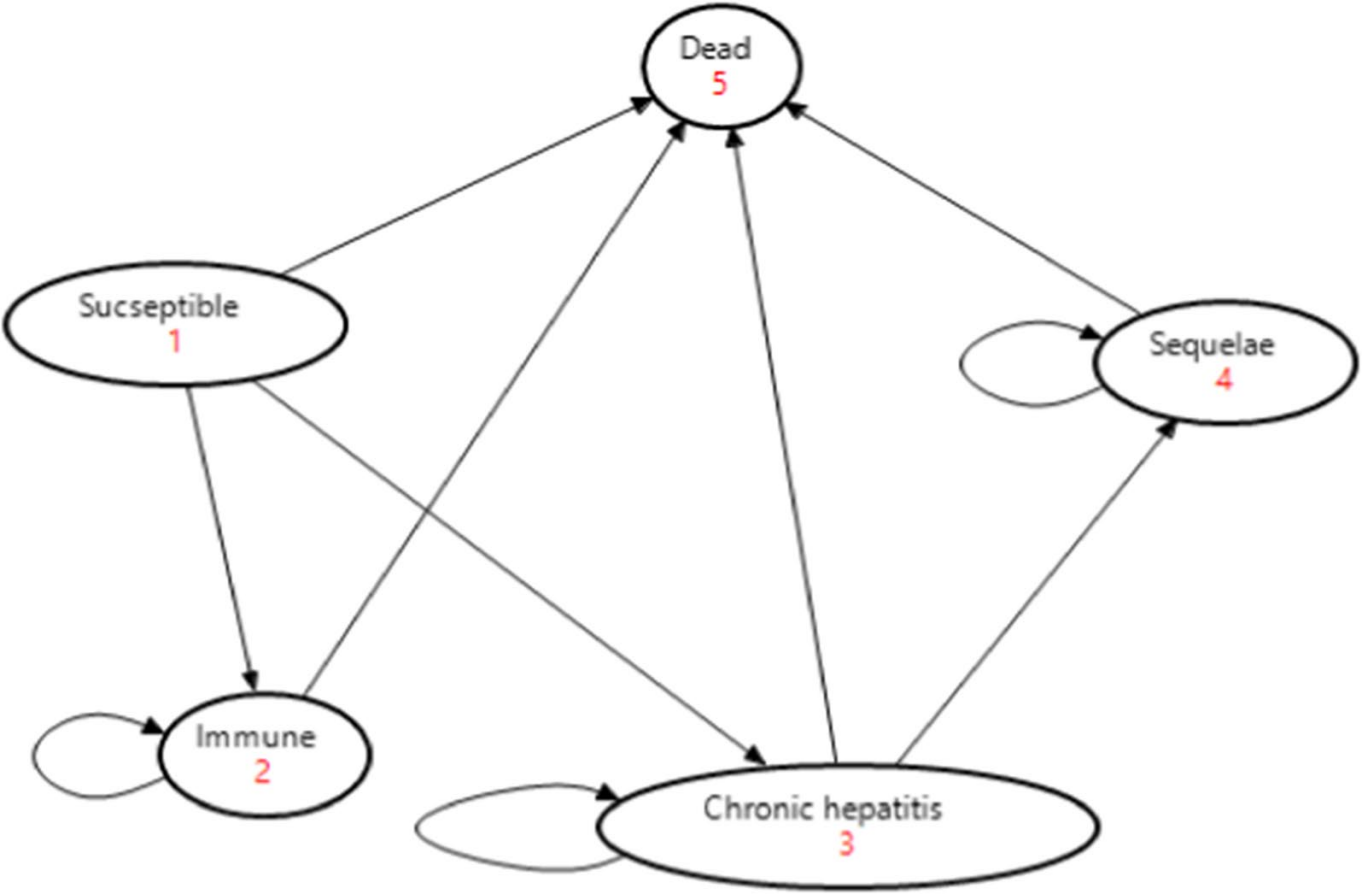
Model of hepatitis B virus disease progress and consequence

The model began with a mean age of average Ethiopian HCWs (32 years) and included 33 cycles (1-year cycle length) to simulate the disease process over the worker's lifetime (65 years as an average Ethiopian life expectancy). The model of HBV disease progress depicted in Fig. 1 indicates any susceptible individual may stay in a “susceptible state” without acquiring an infection. Otherwise, get infected by HBV first acutely with a 5–10% chance of progressing to CHB and a 0.5–1% case fatality rate. The other chance for susceptible individuals is to develop self-immunity or get vaccinated for prior infection. Any person who has immunity could stay in an “immune” health state. The other health state is the “Chronic hepatitis” state. In this health state, an infected person may live as a chronic carrier or get treatment and then progress to a healthy (“immune”) state. When any abnormality follows or results from a CHB infection such as liver permanent damage (cirrhosis, Ascites, and hepatocellular carcinoma), the individual enters into “Sequelae” health state. Despite this, any health state has a probability of entering an absorbing state (death).

### Assumptions

Hepatitis B vaccines are available in different formulations for an instant in combination with other vaccines, such as diphtheria–tetanus–pertussis (DTP), Haemophilus influenza type b (Hib), and Inactivated Poliovirus (IPV). However, in this economic evaluation, only the monovalent HepB vaccine was considered, since it is presently implemented, with an effectiveness/protection rate of 90% in Ethiopia [8, 20]. The term ‘immunized’ was considered for HCWs receiving a complete series of 3 doses of HepB vaccine within a 6-month duration. All HCWs who did not receive the vaccination before were assumed to be eligible for vaccination without being screened for HBV. Our model did not account for the spread of HBV among HCWs or between healthcare facilities because of the lack of country-specific data. Because acute hepatitis is essentially a self-limiting infection, the cost of treatment is not considered in this economic evaluation. Only supportive care, such as resuscitation and symptomatic treatment, was considered for patients with liver complications, such as hepatocellular carcinoma and cirrhosis. In this study, it was assumed that vaccination results in the lifelong immunization of HCWs (up to 65 years).

### Model input

#### Effectiveness data

As stated in Table 2, the data on the CHB disease progression, the disease's natural history, the effectiveness of vaccines in preventing infections, the effectiveness of treatments in slowing the disease's progression towards sequelae, and the health outcome were gathered for this study from a variety of sources of literature and guidelines [5, 6, 16].

**Table 2:** Various probability estimations of hepatitis B disease progression obtained from published follow-up studies

Health event incidence/transition	Probability	Range	Data source
Incidence of hepatitis B infection without vaccination among HCWs	0.6	0.4–0.8	[15]
The transition from acute hepatitis B to chronic HepB	0.05	0.05–0.1	[5]
Progression of untreated HepB to sequelae (cirrhosis, HCC)	0.05	0.02–0.054	[1]
Death due to HepB infection	0.0106	0–0.0106	
Incidence of sequelae chronic following HepB treatment	0.0175	0.017–0.022	[5, 8]
Death due to HepB complication	0.2	0.15–0.25	[22]

*HCC* hepato-cellular carcinoma, *HCWs* Healthcare workers, *HepB* hepatitis B

#### Cost data

The cost of three series doses of the HepB vaccine was determined for every susceptible healthcare worker who is unvaccinated, which makes up about 86% of the current active healthcare workforce (Table 3). One dose of the monovalent HepB vaccine costs USD 17.8, and expenses for administering the vaccine were also taken into consideration. It was estimated that each HCW would spend USD 57 on the vaccine and its administration. For HepB treatment, generic Tenofovir 300 mg tablet is given as a once-daily dose (USD 0.147 per tablet for 365 days) with an optional Furosemide 20 mg tablet (USD 0.1115 per tablet for 30 days) as prophylaxis to sequelae, and its cost was estimated at USD 57 per year/cycle length [5]. The cost covers the majority of necessary laboratory tests, such as liver function tests (ALT, AST), HBsAg, albumin, BIR, and BUN. However, the HBV DNA test, which is the most expensive and inaccessible test, was not included in the cost analysis. The hospitalization and symptomatic treatment costs (USD 189) for patients who experience sequelae such as cirrhosis, ascites, portal hypertension, and hepatocellular carcinoma were also added to the cost of laboratory tests and treatment. Liver transplant cost for patients with liver failure was not estimated, since it is not applicable in Ethiopia.

**Table 3:** Estimated individual annual hepatitis B vaccination cost and hepatitis B disease management cost

Service	List of items consumed/services provided	Total cost per year/cycle length
Vaccination	Hepatitis B, glove, syringe	USD 57
Hepatitis B treatment	TDF 300 mg daily ± diuretics	USD 57
Laboratory investigation	HBsAg ALT, AST, INR, BUN, Albumin, Creatinine	USD 88.56
Hospitalization cost	Symptomatic treatment and bed admission	USD 189

*ALT* alanine amino transferase, *AST* aspartate amino transferase, *HBsAg* hepatitis B surface antigen, *INR* international normalized ratio, *TDF* tenofovir

Most of the information regarding the cost of HepB vaccination and CHB disease management was obtained from pharmacists, nurses, and physicians who actively work in TASH at the department of Gastroenterology, pharmacy, and internal medicine. Indirect costs, transportation costs, capital item costs (such as cold chain storage, deep freezer vans, or vehicles), and other operational costs related to logistics were not included. Finally, the upfront cost (such as fryers/advertisement) to increase vaccine coverage was not considered due to a lack of data. The cost of the three series of vaccine doses and their administration costs (onetime costs) were considered at the beginning cycle of the simulation. Whereas the other costs (such as treatment, laboratory tests, and hospitalization costs) were considered over the lifetime of the healthcare workforce (Table 3).

#### Model output

An output of this economic evaluation includes incremental cost, incremental effectiveness, and its ICER per LYs gained calculated. Since Ethiopia does not have a standardized Cost-Effectiveness Threshold (CET), the World Bank report of Ethiopia's Gross Domestic Product (GDP) per capita (USD 783) was used to determine CET. Accordingly, GDP per capita was multiplied by one or three to obtain an estimated CET of USD 2,349 per QALY gain.

#### Sensitivity analysis

Deterministic sensitivity analysis was employed to manage some uncertainty relating to methodological assumptions (such as time, cost, and effectiveness) variation of distinctive parameters and heterogeneity among the healthcare workforce. In addition, a tornado diagram was also used to illustrate the plausible ranges of parameters obtained from published literature and guidelines of the country.

## Results

**Fig. 2:**
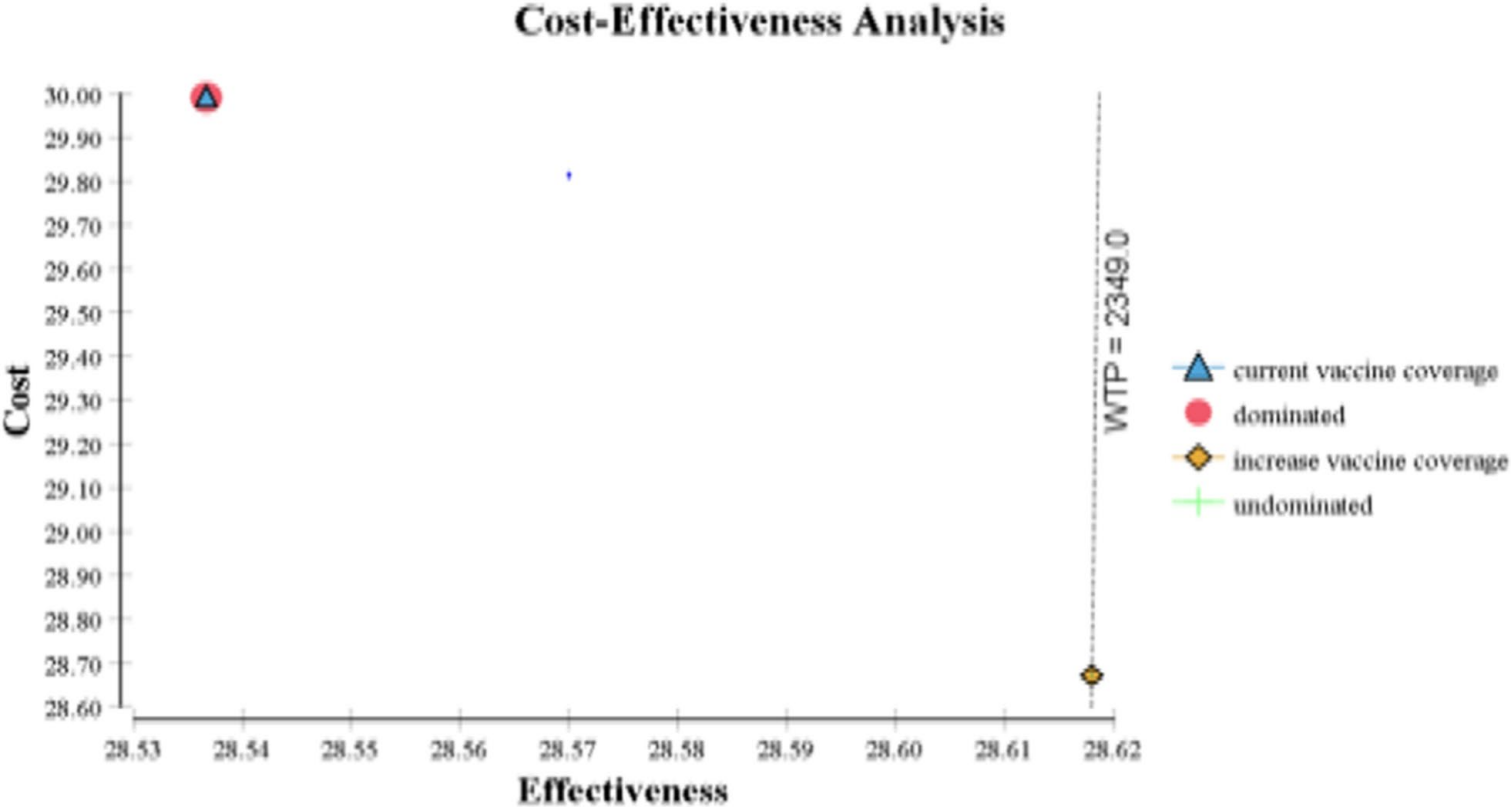
Cost-effectiveness analysis of increasing hepatitis B vaccination coverage

The expanded 80% coverage strategy was found to have higher total life year gains (28.62 LYs) with a lower investment of USD 28.67 compared to the current non-mandatory vaccination coverage (14%) program which is more expensive (USD 29.99) and has lower total life years (28.54 LYs). The expanded coverage averts the cost of USD 1.32 with the negative ICER value (USD-16.5 per LYs gain) (Table 4). This shows that expanding the vaccination program saves USD 16 compared to the current non-mandatory vaccination strategy, confirming its dominance and greater health benefits. The final average or expected costs of the two alternatives were less than the estimated costs which could be determined by the probability of getting the treatments such as Tenofovir or other treatments (due to unavailability and/or unaffordability issues). In general, increasing vaccination coverage to 80% dominated the current coverage through the non-mandatory vaccination strategy (Fig. 2).

**Table 4:** Base-case results from cost-effectiveness analysis of expanding HepB vaccination coverage vs current coverage (June 2019)

Strategy	Life Years	Cost	Incremental LYs	Incremental cost	ICER
Current HepB vaccination coverage (14%)	28.54	29.99	–	–	–
Expanding HepB vaccination coverage (80%)	28.62	28.67	0.08	USD-1.32	− 16.5

*ICER* incremental cost-effectiveness ratio, *USD* United States Dollar

**Fig. 3:**
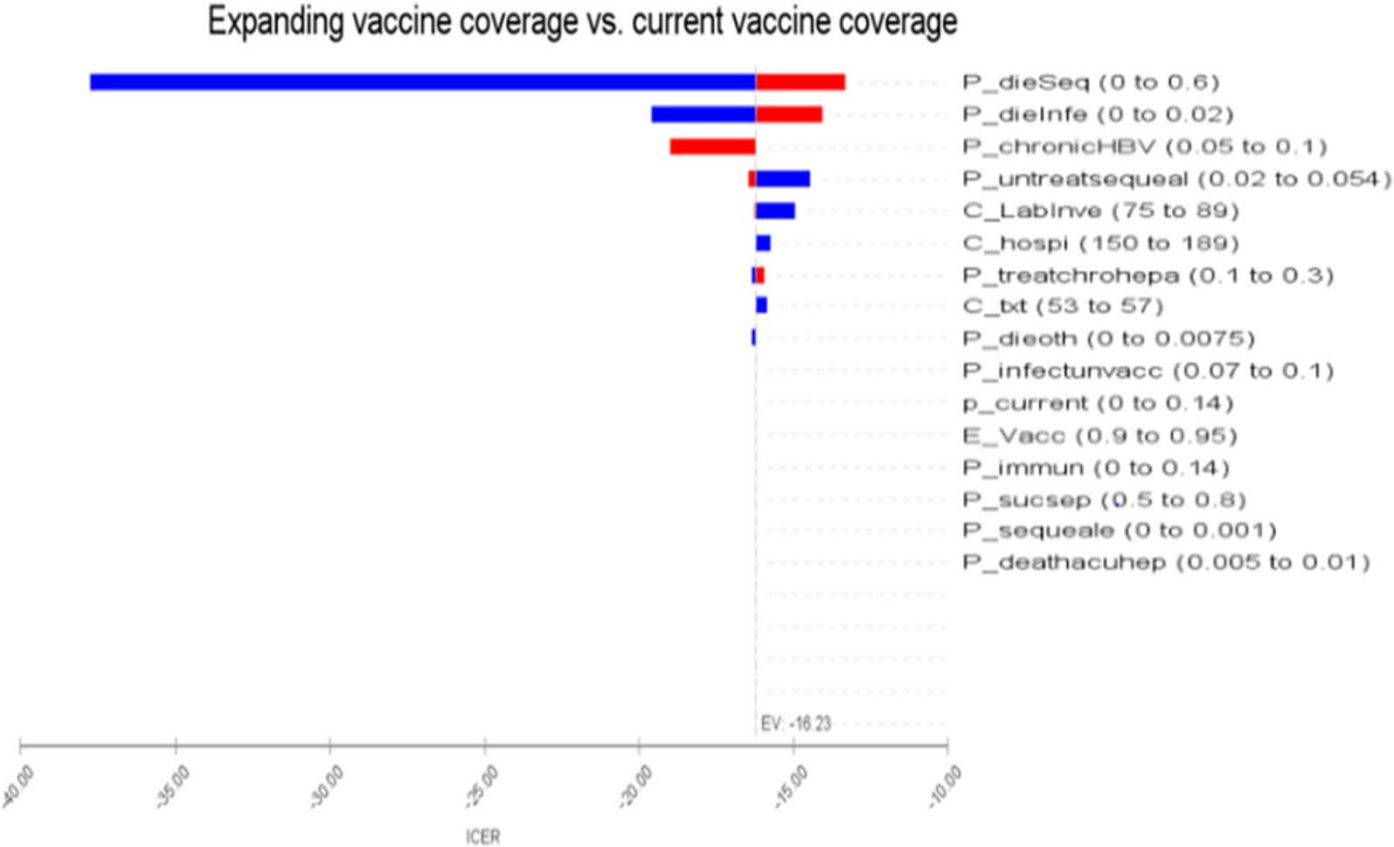
Tornado diagram of current hepatitis coverage vs increasing current hepatitis coverage*. *ICER: incremental cost-effectiveness ratio; P_dieSeq: probability of die due to sequale; P_dieinfe: probability of die due to Hepatitis B virus infection; P_ChroniHBV: probability of chronic hepatitis B virus progression; P_untreatsquale: probability of untreated HCWs with sequale; C_labinve: cost of lab investigation; C_hosp: cost of hospitalization; P_treatchrohepa: probability of treated HCWs with chronic hepatitis; C_txt: cost of CHB treatment with tenofovir and optionally with diuretics as a prophylaxis for squelae; P_dieoth: probability of die due to other causes; P_infectunvacc: probability of infection in unvaccinated HCWs; P_current: probability of current vaccine coverage; E_vacc: effectiveness of Vaccine; P_immun: probability of developing immunity; P_Sucsep: probability of susceptibility; P_sequelae: probability of progressing to sequelae; P_deathacuhep: probability of death due to acute hepatitis

One-way sensitivity analysis provides that the current vaccine coverage was still dominated by an increase in the risk of infection among unvaccinated individuals. For instance, the result of the ICER increased from − 0.9 to − 0.81 when the risk of infection among unvaccinated is increased. Accordingly, the cost has increased from USD 1.54–2.04, while the effectiveness had decreased from − 0.09 to − 0.13 as the chance of infection is raised with the current vaccine coverage. The additional cost incurred was due to post-infection treatment costs. Similarly, the tornado results revealed that the main parameters that may have an impact on the ICER value are the probability of death from sequelae, death from HepB infection, progression of untreated CHB to sequelae, cost of lab investigation, and probability of treating CHB (Fig. 3). The ICER value was increased by changing the cost parameters within the acceptable range and becomes decreased or increased with the varying probabilities of death due to sequelae and death due to HBV infection.

**Fig. 4:**
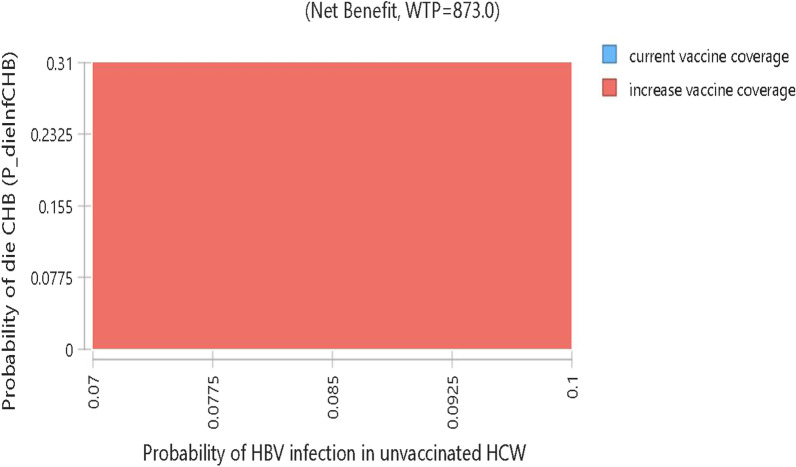
Two-way sensitivity analysis of infection among unvaccinated vs death due CHB infection

According to the two-way sensitivity analysis, increasing vaccine coverage enhances total LYs gain irrespective of the probability that CHB will result in death or the likelihood of HBV infection in unvaccinated individuals. Figure 4 shows that the current coverage strategy (blue) is dominated or covered by the expanded coverage one (red). In general, increasing vaccination coverage to 80% dominated the current coverage through the non-mandatory vaccination strategy (Fig. 2).

## Discussion

The foremost aim of this economic evaluation was to compare the cost-effectiveness analysis of increasing vaccination by 80% vs non-mandatory current coverage (14%). The costs per healthcare worker and the benefit in health in terms of LY gains were calculated. This analysis used a variety of secondary data, primarily effectiveness data, that were taken from published articles. In addition, TASH was used to gather estimated cost data for CHB treatment and vaccination [1, 6, 25]. For every LY gained over a lifetime horizon, the new expanded coverage (80%) strategy costs USD 16.5 less than the coverage by the current vaccination (14%) strategy. Therefore, increasing vaccination coverage is essential, since it is a less expensive and highly effective strategy to combat HepB infection in healthcare personnel. Deterministic sensitivity analyses showed that varying the probability of different variables of cost and effectiveness as the new strategy significantly dominates the current program (Fig. 2). While tornado result indicates that some variables impact the ICER value (Fig. 3). The Cost-effectiveness threshold of USD 2,349, which was three times Ethiopia's GDP, was used to check the affordability of the new strategy. Therefore, since the ICER is negative, implementing the new expanding strategy may have more advantages and be more cost-effective for FMOH to vaccinate its HCWs.

In general, increasing vaccination rates among HCWs to 80% from the current coverage (14%) was found to be more cost-effective in Ethiopia. Thus, the HepB monovalent vaccine offers HCWs lifetime protection with a 90% effectiveness rate in preventing infection [18]. However, the slight reduction of the prevalence (from 7.4% to 6%) from 2016 to 2019 among HCWs in Ethiopia may be associated with lower vaccination coverage through the current non-mandatory strategy [15]. Therefore, the new strategy (expanding to 80%) is highly suggested in Ethiopia, because it was more effective and saves the additional costs that the current coverage (14%) strategy would have required. For example, adopting a mandatory vaccination strategy could be a strategy to increase coverage. A mandatory vaccination strategy was adopted in several nations, including Australia, Austria, Belgium, Canada, the Czech Republic, Germany, Greece, Holland, Ireland, Italy, Poland, Slovakia, Sweden, and the United States, and they also reported that it was effective in lowering the rate of HBV infection among HCWs [26–28]. Economic evaluations are not a common practice in Ethiopia's healthcare system, so conducting such an analysis would help decision-makers and public healthcare payers in allocating their limited healthcare resources cost-effectively. Therefore, public policy makers such as Ethiopian FMOH recommended adopting and applying the expanded HepB vaccination strategy for HCWs across the country.

### Study limitation

This economic analysis has some limitations, particularly concerning costing variables. We did not include viral transmission rates among study participants due to a lack of country-specific data, which might have underestimated the benefit of our findings. Indirect costs related to increasing vaccination coverage, such as vaccine transport and cold chain equipment cost, were not included. Even though the expansion of vaccination coverage across the country’s healthcare facilities requires a high investment of capital items. Therefore, we recommend for future studies include those costs. On the other hand, the cost of acute HepB treatment and the cost of a liver transplant for patients with liver failure were not explicitly determined or included. Direct non-medical costs such as transportation costs and lost wages resulting from illness for every member of the healthcare workforce were also excluded. Although this study places a lot of emphasis on the need to reduce occupational exposure to HepB infection in HCWs by providing vaccines, it does not estimate the cost of screening before vaccination and it makes the methodological supposition that all HCWs are susceptible and eligible for vaccination. Despite all these limitations, efforts have been made to use reliable data by gathering cost data from practicing physicians and other healthcare professionals in TASH. A sensitivity analysis was also performed to test the model's robustness and determine whether it is consistent with the base-case analysis or not.

## Conclusions

From this economic evaluation, it is possible to conclude that increasing current vaccine coverage from 14% to at least 80% among all Ethiopian healthcare workforces was the most cost-effective strategy. As long as expanding hepatitis B vaccine coverage does not incur additional costs and in the meantime, it improves the healthy life of susceptible HCWs and Ethiopian FMOH should work on its implementation.

## Abbreviations

ALT: Alanine amino transferase
AST: Aspartate amino transferase
BIR: Bilirubin
BUN: Blood urea nitrogen
CHB: Chronic hepatitis B
DNA: Deoxyribo-nucleic acid
ELY: Expected life year
EPSA: Ethiopian pharmaceutical supply agency
FMOH: Federal Ministry of Health
GDP: Gross domestic product
HBsAg: Hepatitis B surface antigen
HBV: Hepatitis B virus
HCC: Hepato-cellular carcinoma
HepB: Hepatitis B
HCW: Healthcare worker
ICER: Incremental cost-effectiveness ratio
INR: International normalized ratio
Lys: Life years
QALY: Quality adjusted life year
TASH: Tikur Anbesa Specialized Hospital
USD: United States Dollar
USA: United States of America
WHO: World Health Organization

## Declarations

### Availability of data and materials

All data generated and analyzed during this study are included in this published article and are publicly available.
